# Construction and Immunogenicity Comparison of Three Virus-Like Particles Carrying Different Combinations of Structural Proteins of Avian Coronavirus Infectious Bronchitis Virus

**DOI:** 10.3390/vaccines9020146

**Published:** 2021-02-11

**Authors:** Yu Zhang, Yuan Yuan, Li-Hua Zhang, Dan Zhu, Lu Wang, Lan-Ping Wei, Wen-Sheng Fan, Chang-Run Zhao, Yan-Jing Su, Jian-Qi Liao, Lu Yong, Tian-Chao Wei, Ping Wei, Mei-Lan Mo

**Affiliations:** College of Animal Science and Technology, Guangxi University, Nanning 530004, China; 1818303038@st.gxu.edu.cn (Y.Z.); 1618305016@alu.gxu.edu.cn (Y.Y.); 1418305025@alu.gxu.edu.cn (L.-H.Z.); 1818393058@alu.gxu.edu.cn (D.Z.); 1818393037@alu.gxu.edu.cn (L.W.); 1818403007@st.gxu.edu.cn (L.-P.W.); 1618403001@st.gxu.edu.cn (W.-S.F.); 1718305023@alu.gxu.edu.cn (C.-R.Z.); 1634130106@alu.gxu.edu.cn (Y.-J.S.); 2018302019@st.gxu.edu.cn (J.-Q.L.); 1918302036@st.gxu.edu.cn (L.Y.); weitianchao@gxu.edu.cn (T.-C.W.)

**Keywords:** infectious bronchitis virus, virus-like particles, construction, immunogenicity

## Abstract

Infectious bronchitis virus (IBV) poses massive economic losses in the global poultry industry. Here, we firstly report the construction and immunogenicity comparison of virus-like particles (VLPs) carrying the S, M and E proteins (SME-VLPs); VLPs carrying the S and M proteins (SM-VLPs); and VLPs carrying the M and E proteins (ME-VLPs) from the dominant serotype representative strain GX-YL5 in China. The neutralizing antibody response induced by the SME-VLPs was similar to that induced by the inactivated oil vaccine (OEV) of GX-YL5, and higher than those induced by the SM-VLPs, ME-VLPs and commercial live vaccine H120. More importantly, the SME-VLPs elicited higher percentages of CD4^+^ and CD8^+^ T lymphocytes than the SM-VLPs, ME-VLPs and OEV of GX-YL5. Compared with the OEV of GX-YL5, higher levels of IL-4 and IFN-γ were also induced by the SME-VLPs. Moreover, the mucosal immune response (sIgA) induced by the SME-VLPs in the tear and oral swabs was comparable to that induced by the H120 vaccine and higher than that induced by the OEV of GX-YL5. In the challenge experiment, the SME-VLPs resulted in significantly lower viral RNA levels in the trachea and higher protection scores than the OEV of GX-YL5 and H120 vaccines, and induced comparable viral RNA levels in the kidneys, and tear and oral swabs to the OEV of GX-YL5. In summary, among the three VLPs, the SME-VLPs carrying the S, M and E proteins of IBV could stimulate the strongest humoral, cellular and mucosal immune responses and provide effective protection, indicating that it would be an attractive vaccine candidate for IB.

## 1. Introduction

Infectious bronchitis (IB) is an acute respiratory viral disease of chickens and caused by infectious bronchitis virus (IBV), a member of the coronavirus family. Chickens of all ages and breeds are susceptible to IBV [1]. IBV infection damages the integrity of the respiratory mucosa in chickens and increases the susceptibility to secondary infections of mycoplasma and *Escherichia coli*, leading to higher mortality. IBV infection also permanently destroys the oviducts of immature females and pullets, resulting in limited egg production for a long time and birds that fail to come into production (false layers) [2]. In addition, the kidneys, alimentary tract, and muscles are injured by IBV infection, which causes a decline in weight gain, egg production, eggshell quality and feed efficiency [3]. Therefore, IB causes huge economic losses to the poultry industry worldwide.

IBV contains four structural proteins that are the spike (S), envelope (E), membrane (M) and nucleocapsid (N) proteins [1]. The S protein is prone to variation. It is the most significant component of the viral envelope because it carries serotype-specific and virus-neutralizing epitopes, mediates cell attachment, and induces membrane fusion and receptor binding [4,5,6]. The M protein, which is the most abundant structural protein, mediates virus particle assembly and budding [7,8]. The E protein plays a crucial role in envelope formation [9]. Interactions between the M and E proteins have been considered to be the minimum requirements for the formation of coronavirus virus-like particles (VLPs) [10].

Vaccination is still the main effective means for the preventing and controlling of the disease. However, traditional inactivated and attenuated vaccines cannot provide complete and effective protection due to the lack of serotype matching between the vaccine and field variant strains [11,12,13]. Live-attenuated vaccines produce protective cellular and mucosal immunity. However, these types of vaccines may pose the risks of mutation and recombination with other vaccines or field strains [14,15,16]. Meanwhile, it is also possible that there is a risk of them returning to virulent form, causing disease in normal individuals [17,18]. Inactivated vaccines are safe and effective in stimulating humoral immune responses, but fail to establish long-term immunity and induce strong cellular and mucosal immunity [19,20,21]. Therefore, there is an urgent need for the development of safe and efficient IBV vaccines.

Despite the widespread application of IBV vaccines, IB has been a continuing problem in the poultry industry. Many IBV variant strains have been isolated and identified. Studies have shown that the genotypes and serotypes of the majority of IBV isolates are different from those of traditional vaccine strains in China [22,23,24]. In addition, there are multiple genotypes and serotypes, among which exist dominant genotypes and serotypes of IBV strains [25,26,27]. Our recent studies showed that none of the H120, 4/91 and LDT3-A commercial attenuated vaccines could provide complete protection against the dominant serotype IBV epidemic strain in southern China [28]. Thus, it is of great significance to develop a new IBV vaccine that is safe, efficient and matched with the dominant serotype of the IBV epidemic strain for the prevention and control of IB in China.

Among new vaccines, the VLP vaccine is considered one of the most promising due to its high safety and good immunogenicity [29]. VLPs are automatically assembled from one or more structural proteins of the virus, mimicking the conformation and organization of authentic native viruses and lacking viral genomes [30]. Owing to the lack of viral genomes and capacity to replicate autonomously, they are a safe vaccine candidate [30]. Moreover, VLPs stimulate the body to produce effective immune responses [31,32]. At present, VLPs of severe acute respiratory syndrome coronavirus (SARS-CoV) and Middle East respiratory syndrome coronavirus (MERS-CoV) are the most studied VLPs of coronaviruses. SARS-CoV VLPs assembled from the S, E and M proteins were effective in eliciting systemic and mucosal immune responses [33]. MERS-CoV VLPs containing the S, E and M proteins had excellent immunogenicity and could induce robust levels of specific humoral and cell immunity [34,35]. However, the proteins required for the formation of coronavirus VLPs varied in different reports. Some researchers showed that the M and E proteins were needed for the assembly of human SARS-CoV VLPs [36]. Some reported that the M, E and N proteins were required [37].

It is unclear how many combinations of structural proteins can assemble into IBV VLPs. To date, there have been few studies on IBV VLPs. A previous study showed that IBV VLPs, containing the M and S proteins of the H120 strain, could elicit humoral immunity comparable to that achieved with the H120 inactivated vaccine and a cellular immune response higher than that induced by the H120 inactivated vaccine [38]. IBV VLPs carrying the M, E and recombinant S proteins of the M41 strain were comparable to that of the inactivated M41 viruses in eliciting IBV-specific antibodies and neutralizing antibodies [39]. Chimeric VLPs containing the S glycoprotein of the H120 strain induced significantly higher S1-specific antibody and neutralization antibody levels than inactivated H120 virus in chickens [40]. Recently, novel chimeric VLPs bearing the recombinant spike protein (rS) and M protein of the IBV M41 strain, and the recombinant fusion protein (rF) of Newcastle disease virus (NDV), were highly immunogenic and could provide complete protection against an IBV or NDV virulent challenge [41]. However, these studies had some drawbacks. Firstly, all the VLPs observed in these studies were targeted at a Mass-type IBV strain, not at a local dominant epidemic strain. Secondly, the effects of VLPs on mucosal immunity were not analyzed in these studies, and the immune protection was seldom observed. Thirdly, the immune response and protection afforded by VLPs formed with different combinations of structural proteins of IBV were not compared in these studies.

At present, there have been no reports on comparisons of the immunogenicity and protection efficiency among SME-VLPs, SM-VLPs and ME-VLPs, carrying S, M and E proteins; S and M proteins; and M and E proteins, respectively. In this study, three VLPs, SME-VLPs, SM-VLPs and ME-VLPs, from the dominant serotype representative strain GX-YL5 in southern China were constructed. It is worth mentioning that southern China is the region with the largest production of local breeds of chickens, about 5 billion annually, in the country. In order to screen the VLP vaccine candidates for IB, the immunogenicity and protection efficacy of the three VLP vaccines were evaluated and compared in specific-pathogen-free (SPF) chickens. This is the first report on the construction and immunogenicity comparison of three VLPs carrying different combinations of structural proteins of IBV. This study provides a basis for the exploration of VLP vaccine candidates for IB.

## 2. Materials and Methods

### 2.1. Cells and Viruses

Sf9 insect cells were cultured in serum-free Sf-900TM II SFM (GIBCO, Grand Island, NY, USA) and SIM SF Sf9 (Sino Biological Inc., Beijing, China) at 27 °C. The virulent GX-YL5 strain (accession number, HQ 848267.1) of IBV was the dominant serotype representative isolate and had a heterologous serotype compared to the H120 vaccine strain [25].

### 2.2. Generation and Analysis of Recombinant Protein Expression

The S, M and E genes of the GX-YL5 strain were amplified by reverse transcription polymerase chain reaction (RT-PCR) and subcloned into the pFastBac^TM^/HBM-TOPO vector (Invitrogen, Waltham, MA, USA) to obtain the recombinant transposon vectors pFastBac^TM^/HBM-TOPO-S, pFastBac^TM^/HBM-TOPO-M and pFastBac^TM^/HBM-TOPO-E, respectively. The three recombinant transposon vectors were chemically transformed into competent *Escherichia coli* DH10Bac cells to obtain the recombinant bacmids rHBM-S, rHBM-M and rHBM-E by blue/white selection and sequencing using M13 primers. The purified recombinant bacmids were transfected into sf9 cells with Cellfectin reagent following the manufacturer’s instructions (Invitrogen) to obtain recombinant baculovirus rHBM-S, rHBM-M and rHBM-E. The three recombinant S, M and E proteins were identified by immunofluorescence staining analysis and Western blotting. For immunofluorescence staining analysis, the expression of protein was detected using an anti-His mouse monoclonal antibody (TransGen Biotech, Beijing, China) at a dilution of 1:200 and FITC-conjugated goat anti-mouse IgG secondary antibody (1:400 dilution, Bioss, Beijing, China). Cell nuclei were stained with DAPI (Beyotime Biotech, Beijing, China). Chicken polyclonal antiserum (1:2000 dilution) was used as the primary antibody, and an HRP-conjugated goat anti-chicken IgG (1:5000 dilution, TransGen Biotech, Beijing, China), as the secondary antibody for the verification of the three recombinant proteins by Western blotting. The Image J software was applied to analyze the quantification of the intensity of each protein band, and the results are presented as the ratios of the proteins of interest to an internal reference protein.

### 2.3. Preparation and Purification of VLPs

Three IBV VLPs, SME-VLPs, SM-VLPs and ME-VLPs, were prepared by co-infection with three different combinations of recombinant baculovirus (rHBM-S, rHBM-M and rHBM-E; rHBM-S and rHBM-M; rHBM-M and rHBM-E) in suspension-cultured Sf9 cells. The cell culture supernatants were collected and ultracentrifuged at 80,000× *g* for 2 h at 4°C. After the sediments were resuspended in PBS, the solutions were loaded on a discontinuous sucrose gradient of 20%, 40% and 60% sucrose and ultracentrifuged at 80,000× *g* for 3 h at 4°C. The VLPs at the interface between the 40% and 60% sucrose were collected and pelleted by ultracentrifugation at 80,000× *g* for 2 h at 4 °C.

### 2.4. Identification of Virus-Like Particles

Three VLPs were identified by Western blotting as previously described [42]. An anti-His mouse monoclonal antibody (1:2000 dilution, TransGen Biotech, Beijing, China) was used as the primary antibody, and an HRP-conjugated goat anti-mouse IgG antibody (1:5000 dilution, TransGen Biotech, Beijing, China), as the secondary antibody. Additionally, the purified VLPs were ascertained by transmission electron microscopy (TEM) and immunoelectron microscopy (IEM). The three purified VLPs were visualized using transmission electron microscopy with negative staining. For immunogold labeling, the SME-VLPs and SM-VLPs were loaded onto carbon-coated grids, respectively. A specific antibody against the IBV strain GX-YL5 S protein (rabbit antiserum prepared by immunizing New Zealand white rabbits, 1:20 dilution) was used as the primary antibody, and a 10 nm-gold-conjugated anti-rabbit IgG (1:25 dilution, Signa Aldrich, Shanghai, China), as the secondary antibody, as previously described [36,43]. The prepared samples were observed under a transmission electron microscope (HITACHI7700, Japan).

### 2.5. Immunization and Challenge

A total of 120 10-day-old specific-pathogen-free (SPF) chickens were randomly divided into 6 groups, with 20 birds per group. The chickens were immunized 2 times (at weeks 0 and 2) with IBV VLPs or other vaccines at two-week intervals. The inoculation dose for the SME-VLP and SM-VLP groups contained 2 µg of S proteins. The ME-VLP group was vaccinated with VLPs containing 2 µg of M proteins. The birds in the GX-YL5 group were immunized with the OEV of GX-YL5 containing 2 µg of S proteins. The birds in the H120 group were immunized with the commercial attenuated H120 vaccine according to the manufacturer’s protocols. A total of 20 birds were immunized with PBS as a negative control. Besides the birds in the H120 group being immunized by the nasal–ocular route, the birds in the other groups were intramuscularly (i.m.) immunized. At 14 days post-vaccination (dpv), booster vaccinations were performed with the same program and doses as the prime vaccinations. At 14 days post-booster (dpb), all the birds from each group were challenged with a 10^6^ median infective dose of the trachea organ ring (TOC-ID50) of the IBV GX-YL5 strain in 0.2 mL by the nasal–ocular route. The animal experiments were approved of by the Animal Care & Welfare Committee of Guangxi University (approval number, GXU2018-026) and were performed in accordance with animal ethics guidelines and approved protocols.

### 2.6. Detection of IBV-Specific Antibodies

Serum samples were collected from the inoculated chickens at 0, 7, 14, 21 and 28 dpv. A commercial enzyme-linked immunosorbent assay (ELISA) IBV antibody test kit (IDEXX Laboratory, Inc., Westbrook, ME, USA) was used to evaluate the IBV-specific antibodies in the sera following the manufacturer’s instructions.

### 2.7. Detection of IBV-Neutralizing Antibody Titers

At 0, 14 and 28 dpv, the IBV-neutralizing antibody titers in the sera from the vaccinated chickens were determined by a trachea organ ring (TOC) neutralization test following our previous description [25]. TOCs were prepared from 20-day-old chicken embryos. The TOC-ID50 of the GX-YL5 strain was examined. For the neutralization test, serum samples were serially diluted two-fold with sterile PBS and mixed with 200 TOC-ID50 of the GX-YL5 strain. The highest serum dilution that could neutralize the virus, which caused ciliostasis and beaten cilia, was the neutralization titer. 

### 2.8. Flow Cytometry

Peripheral blood samples were collected from the wing veins of 10 birds in each group at 0, 7, 14, 21 and 28 dpv. Peripheral blood lymphocytes were isolated from the peripheral blood samples. The samples were diluted with equal volumes of sterile PBS, overlaid onto chicken peripheral blood lymphocyte isolation liquid (Solarbio, Beijing, China) and centrifuged at 500 g for 20 min at room temperature. The peripheral blood lymphocytes at the interface were collected and washed with sterile PBS. A final volume of 0.1 mL was stained with 3 µL of Mouse Anti-Chicken CD3-PE, 2 µL of Mouse Anti-Chicken CD4-PE/CY7 and 2 µL of Mouse Anti-Chicken CD8α-FITC antibodies (SouthernBiotech, Birmingham, AL, USA), respectively. The concentrations of the Mouse Anti-Chicken CD3-PE, CD4-PE/CY7 and CD8α-FITC antibodies were adjusted to 0.3, 0.1, and 1 µg/0.1 mL, respectively. All the samples were assessed for the percentages of CD3^+^, CD4^+^ and CD8^+^ T lymphocytes using a BD AccuriTMC6 Flow Cytometer (Becton, Dickinson and Company, Minneapolis, MN, USA). All the staining reactions were conducted according to the manufacturer’s protocols.

### 2.9. Cytokine Assay

Serum samples were collected from the birds at 0, 14 and 28 dpv. The concentrations of IL-4 and IFN-γ in the sera were determined using ELISA Kits (Cloud-Clone, Wuhan, China) according to the manufacturer’s protocols, respectively.

### 2.10. Detection of Secretory IgA(sIgA)

Tear and oral swabs were collected at 0, 7, 14, 21 and 28 dpv. The sIgA concentrations were evaluated using an ELISA Kit (Cloud-Clone, Wuhan, China). The sIgA antibody detection procedures were completed in accordance with the instructions.

### 2.11. Detection of Virus Loads by Real-Time Quantitative PCR

At 5 days post-challenge (dpc), 10 birds in each group were sacrificed to collect the trachea and kidney tissue samples. The remaining birds were observed until 14 dpc. The tear and oral swabs were collected at 2, 4, 6, 8, 10, 12 and 14 dpc. The RNA in the tracheas, kidneys, and tear and oral swabs was extracted using a commercial AxyPrep^TM^ multisource total RNA miniprep kit (Corning Biotech, Suzhou, China). The viral RNA levels were assessed by real-time quantitative PCR (qPCR) targeting the nucleocapsid (N) gene, as previously described [44].

### 2.12. Ciliostasis Test

Ten birds from each group were euthanized at 5 dpc. Ten tracheal rings from each trachea approximately 1–2 mm thick were cut. The ciliostasis test was conducted, and the protection scores for each trachea was calculated as previously described [45].

### 2.13. Statistical Analysis

All the data are presented as geometric means with 95% CIs. Statistical analysis was performed by a one-way analysis of variance (ANOVA) using the SPSS 24.0 software. P values less than 0.05 (*p* < 0.05) were considered significant, and those less than 0.01 were considered highly significant.

## 3. Results

### 3.1. Expression of S, M and E Proteins in Sf9 Cells

**Figure 1:**
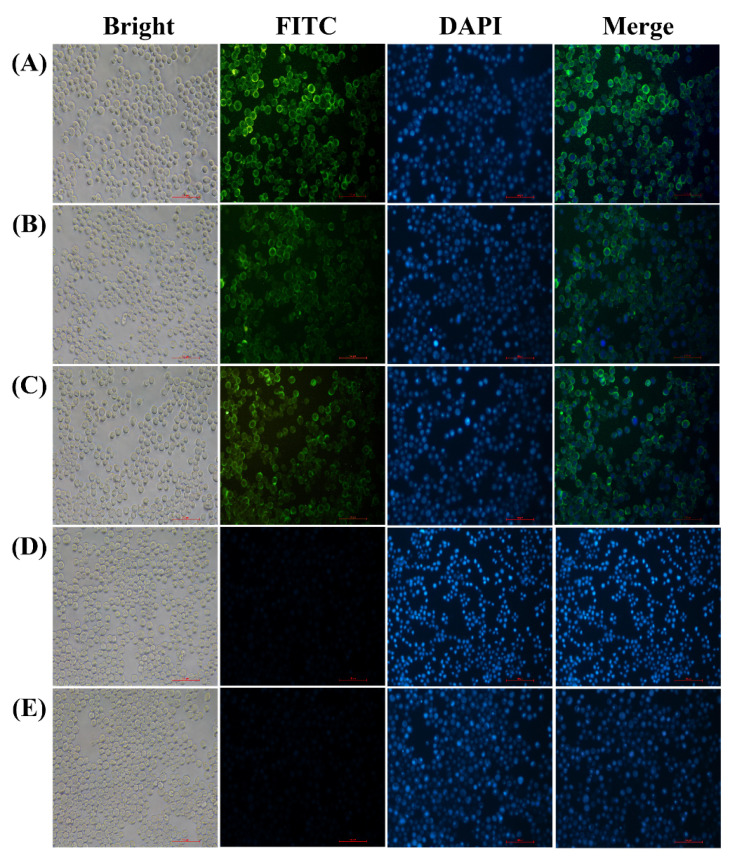
Immunofluorescent staining analysis of M, S and E protein expression in the recombinant baculovirus-infected Sf9 cells at 60 h post-infection (200×). (**A**) Sf9 cells infected with recombinant baculovirus rHBM-S. (**B**) Sf9 cells infected with recombinant baculovirus rHBM-M. (**C**) Sf9 cells infected with recombinant baculovirus rHBM-E. (**D**) Sf9 cells infected with wild-type baculovirus. (**E**) Sf9 cells. FITC was antibody conjugated (green); DAPI was used to stain cell nuclei (blue), and merging signified that FITC merged with DAPI.

**Figure 2:**
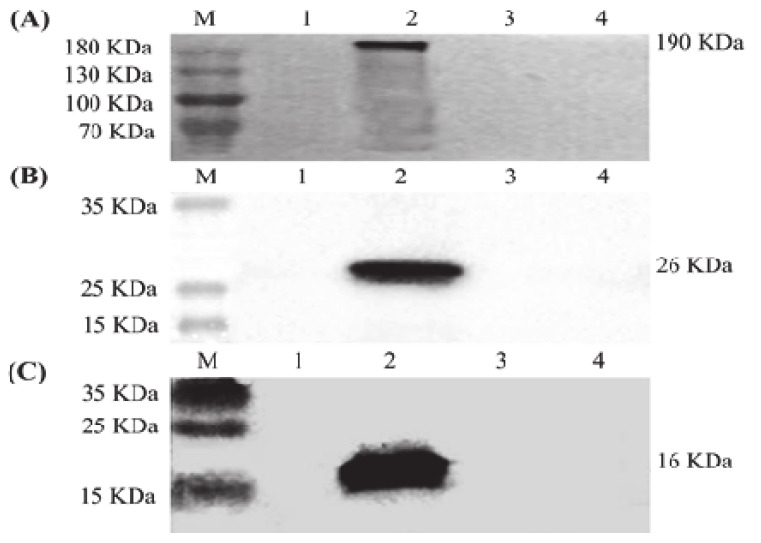
Identification of the recombinant proteins’ expression using Western blotting. (**A**) The S protein expressed by recombinant baculovirus rHBM-S. (**B**) The M protein expressed by recombinant baculovirus rHBM-M. (**C**) The E protein expressed by recombinant baculovirus rHBM-E. Lane M, protein molecular weight marker; Lane 1, culture supernatant; Lane 2, cell lysate; Lane 3, wild-type baculovirus; Lane 4, Sf9 cells.

In order to amplify the baculoviral stocks, Sf9 cells were infected with the recombinant baculovirus rHBM-S, rHBM-M and rHBM-E, respectively. The recombinant proteins S, M and E were determined by immunofluorescence staining analysis and Western blotting. The specific fluorescence signals were detected in the Sf9 cells infected with the recombinant baculovirus rHBM-S (Figure 1A), rHBM-M (Figure 1B) and rHBM-E (Figure 1C). The recombinant S, M and E proteins were mainly expressed near the plasma membranes (Figure 1A–C). No fluorescence signals were detected in the Sf9 cells infected with wild-type baculovirus (Figure 1D) and Sf9 cells (Figure 1E). The specific bands of 190 kDa (Figure 2A), 26 kDa (Figure 2B) and 16 kDa (Figure 2C) proteins were observed in the lysates of the cells infected with the recombinant baculovirus rHBM-S, rHBM-M and rHBM-E, respectively. The results suggest that the recombinant S, M and E proteins were successfully expressed.

### 3.2. Generation and Characterization of IBV VLPs

**Figure 3:**
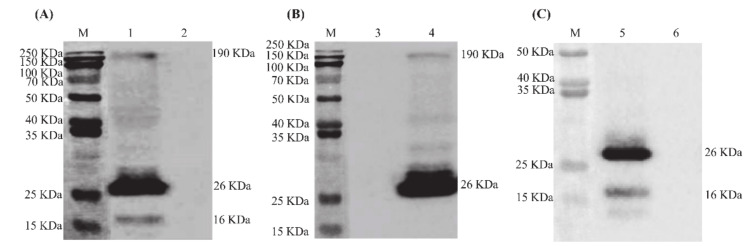
Western blot analysis of virus-like particles (VLPs). (**A**) Western blot analysis of SME-VLPs purified from culture supernatants of cells co-infected with rHBM-S, rHBM-M and rHBM-E. (**B**) Western blot analysis of SM-VLPs purified from culture supernatants of cells co-infected with rHBM-S and rHBM-M. (**C**) Western blot analysis of ME-VLPs purified from culture supernatants of cells co-infected with rHBM-M and rHBM-E. Lane M, protein molecular weight marker; Lane 1, purified VLPs containing S, M and E proteins; Lane 4, purified VLPs containing S and M proteins; Lane 5, purified VLPs containing M and E proteins; Lanes 2, 3 and 6, wild-type baculovirus.

**Figure 4:**
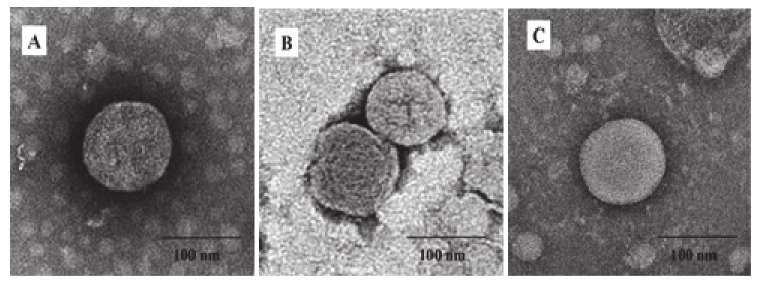
The formation of infectious bronchitis virus (IBV) VLPs identified by TEM. (**A**) Purified SME-VLPs. (**B**) Purified SM-VLPs. (**C**) Purified ME-VLPs. Bar = 100 nm.

**Figure 5:**
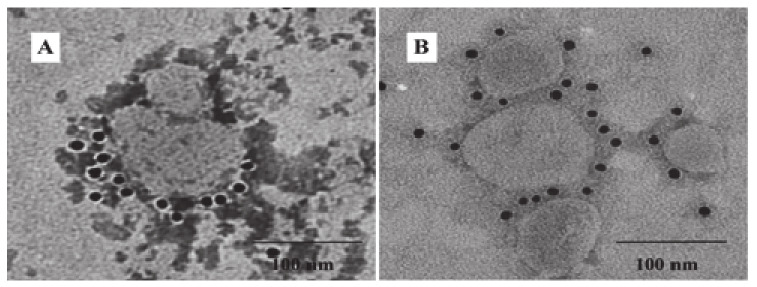
Analysis of the presence of S protein in the IBV VLPs by immunoelectron microscopy (IEM). (**A**) Purified SME-VLPs. (**B**) Purified SM-VLPs. Bar = 100 nm.

The SME-VLPs, SM-VLPs and ME-VLPs were prepared by co-infection with three different combinations of baculovirus in suspension-cultured Sf9 cells. The Western blot results show that the individual corresponding protein bands (S protein, of 190 kDa; M protein, of 26 kDa; E protein, of 16 kDa) were detected in the three VLPs (Figure 3). Circular or polygonal particles with diameters ranging from 80 to 120 nm were observed in all three VLPs under TEM (Figure 4). Black gold-labeled S protein particles on the surfaces of the SME-VLPs and SM-VLPs were observed under IEM (Figure 5). These results revealed that IBV SME-VLPs, SM-VLPs and ME-VLPs were assembled successfully in the insect baculovirus expression system by co-infection.

### 3.3. Humoral Immune Responses in Immunized Chickens

**Figure 6:**
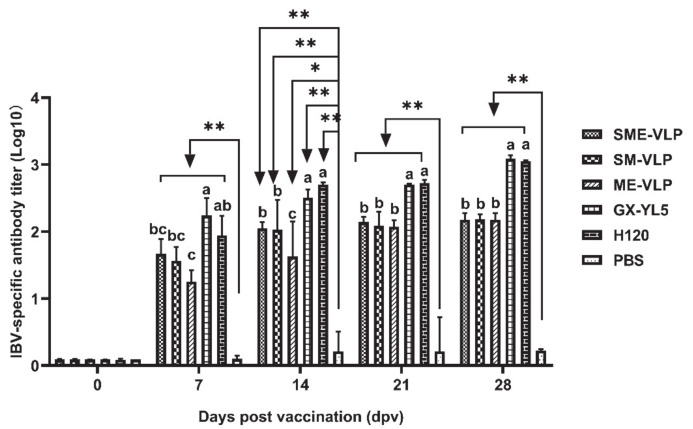
IBV-specific antibody levels in vaccinated chicken sera (0, 7, 14, 21 and 28 days post-vaccination (dpv)) detected by indirect ELISA. Letters (a–c) indicate significant differences among vaccinated groups (*p* < 0.05). ** and * indicate *p* < 0.01 and *p* < 0.05 between vaccinated and nonvaccinated groups, respectively. (*n* = 10 chickens/group.)

At 0, 7, 14, 21 and 28 dpv, the levels of IBV-specific antibodies were analyzed by ELISA. The antibody levels in all the vaccinated groups continued to rise from 7 to 28 dpv (Figure 6). The antibody levels of the immunized groups increased notably at 7, 14, 21 and 28 dpv and were significantly higher than those of the negative control group (*p* < 0.05 or *p* < 0.01). The highest antibodies in each immunized group were observed at 28 dpv. At 14, 21 and 28 dpv, the birds in the GX-YL5 and H120 groups had higher antibody titers than those of the VLP-vaccinated groups (*p* < 0.05), while no significant difference was noted between the GX-YL5 and H120 groups. The antibody levels of the SME-VLP and SM-VLP groups were all significantly higher compared to those of the ME-VLP group (*p* < 0.05) at 14 dpv, but there was no significant difference in the antibody levels among the three VLP groups at 21 and 28 dpv.

**Figure 7:**
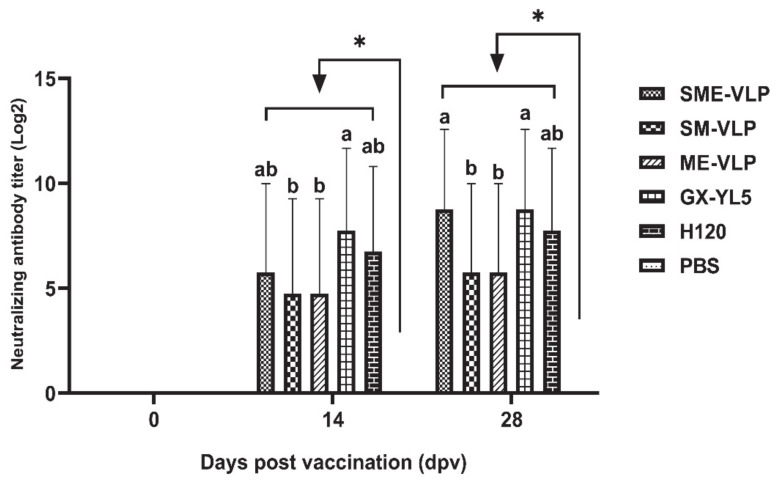
IBV-neutralizing antibodies measured in sera (0, 14 and 28 dpv) by trachea organ ring (TOC) neutralization test. Letters (a,b) indicate significant differences among vaccinated groups (*p* < 0.05). * indicates *p* < 0.05 between vaccinated and nonvaccinated groups. (*n* = 10 chickens/group.)

The IBV-neutralizing antibody titers in the sera were evaluated by TOC neutralization tests at 0, 14 and 28 dpv. The results demonstrate that all the vaccinated groups had higher neutralizing antibody levels than the negative control group at 14 and 28 dpv (*p* < 0.05) (Figure 7). At 14 dpv, the birds in the GX-YL5 group developed higher IBV-neutralizing antibody titers than those of the VLPs- and H120-vaccinated groups (*p* < 0.05). The highest IBV-neutralizing antibody titers in all the immunized groups were observed at 28 dpv. At 28 dpv, the SME-VLP and GX-YL5 groups had the highest and same IBV-neutralizing antibody titers. There were no significant differences in antibody titers among the SM-VLP and ME-VLP groups at 28 dpv.

### 3.4. Cellular Immune Responses in Immunized Chickens

**Figure 8:**
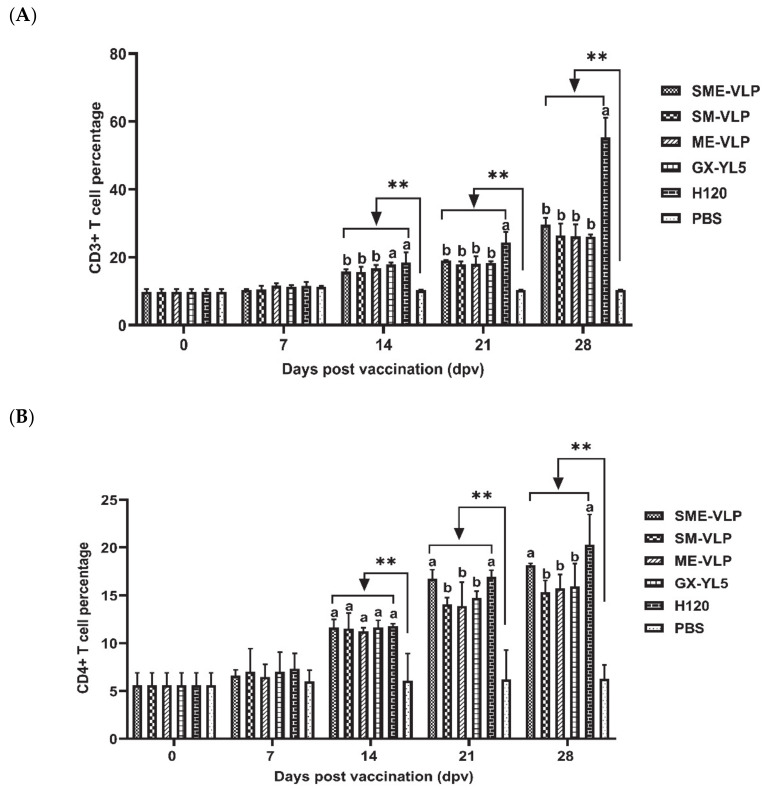

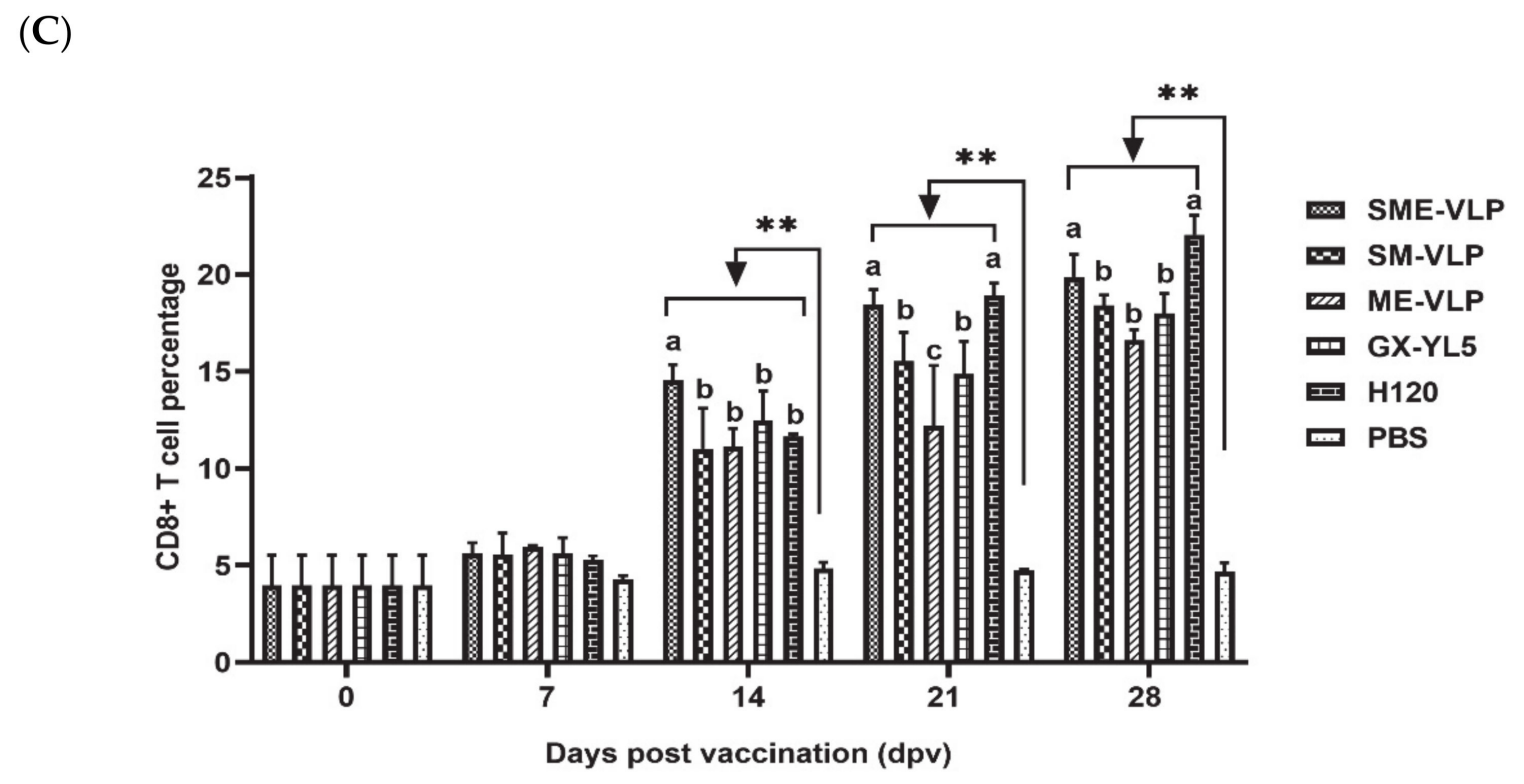
Percentages of CD3^+^, CD4^+^ and CD8^+^ T lymphocytes in peripheral blood of vaccinated chickens. Peripheral blood lymphocytes were isolated from vaccinated chickens at 0, 7, 14, 21 and 28 dpv and analyzed by flow cytometry. (**A**) Percentages of CD3^+^ T lymphocytes. (**B**) Percentages of CD4^+^ T lymphocytes. (**C**) Percentages of CD8^+^ T lymphocytes. Letters (a–c) indicate significant differences among vaccinated groups (*p* < 0.05). ** indicates *p* < 0.01 between vaccinated and nonvaccinated groups. (*n* = 10 chickens/group.)

The results demonstrate that the percentages of CD3^+^, CD4^+^ and CD8^+^ T lymphocytes in all the vaccinated groups were rising at 7 dpv (Figure 8). All the five vaccinated groups were significantly higher than the negative control group (*p* < 0.01) from 14 to 28 dpv. At 21 and 28 dpv, the H120 group presented the highest percentages of CD3^+^ T lymphocytes, which were significantly higher than those of the VLP and GX-YL5 groups (*p* < 0.05). At 21 and 28 dpv, the percentages of CD4^+^ and CD8^+^ T lymphocytes in the SME-VLP and H120 groups were significantly higher than those of the other vaccinated groups (*p* < 0.05), but there were no significant differences between the SME-VLP and H120 groups. No significant differences were noted among the SM-VLP, ME-VLP and GX-YL5 groups at 28 dpv.

**Figure 9:**
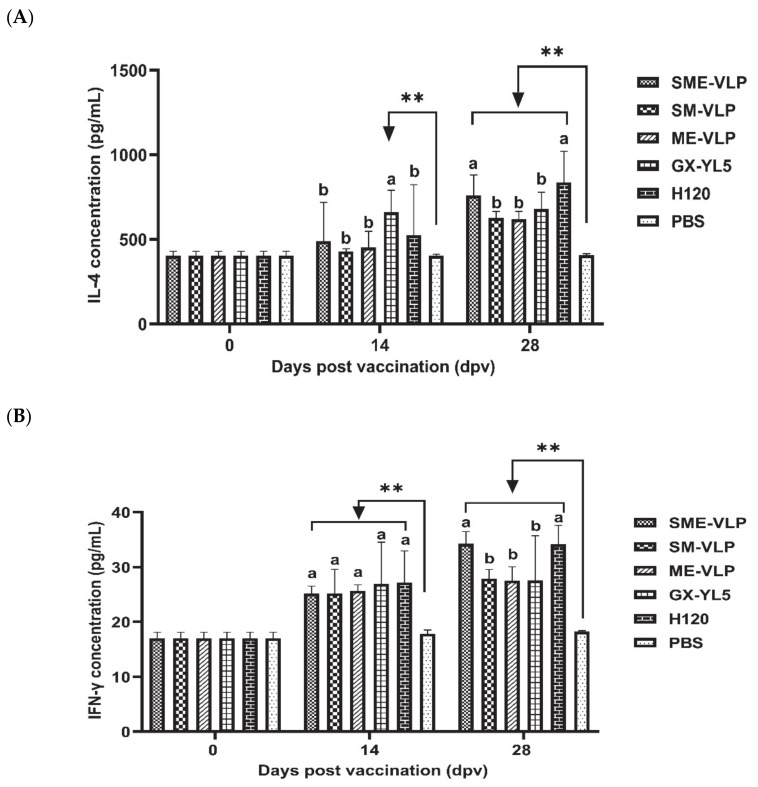
IL-4 and IFN-γ concentrations in the sera of vaccinated chickens at 0, 14 and 28 dpv. (**A**) IL-4 concentrations. (**B**) IFN-γ concentrations. Letters (a,b) indicate significant differences among vaccinated groups (*p* < 0.05). ** indicates *p* < 0.01 between vaccinated and nonvaccinated groups. (*n* = 10 chickens/group.)

The concentrations of IL-4 and IFN-γ in the sera from all the immunized groups were analyzed at 0, 14 and 28 dpv (Figure 9). At 14 dpv, the GX-YL5-vaccinated group had significantly higher IL-4 levels than the nonvaccinated group (*p* < 0.01). At 28 dpv, the concentrations of IL-4 of all the vaccinated groups were significantly higher than those of the nonvaccinated group (*p* < 0.01). In addition, the IL-4 levels of the SME-VLP and H120 groups were significantly higher than those of the SM-VLP, ME-VLP and GX-YL5 groups (*p* < 0.05), and there were no significant differences between the H120 and SME-VLP groups. Higher IFN-γ levels than in the negative control group were successfully induced in all the vaccinated groups on the 14 and 28 dpv. At 14 dpv, the IFN-γ levels in the SME-VLP, SM-VLP and ME-VLP groups were comparable to those of the OEV of GX-YL5 and H120 groups, and they showed no significant differences among the vaccinated groups. At 28 dpv, the IFN-γ levels in the SME-VLP and H120 groups were significantly higher than those of the other immunized groups (*p* < 0.05), but no significant differences were observed between the SME-VLP and H120 groups. There was no significant difference in the IFN-γ levels among the SM-VLP, ME-VLP and GX-YL5 groups at 28 dpv.

### 3.5. Mucosal Immune Responses in Immunized Chickens

**Figure 10:**
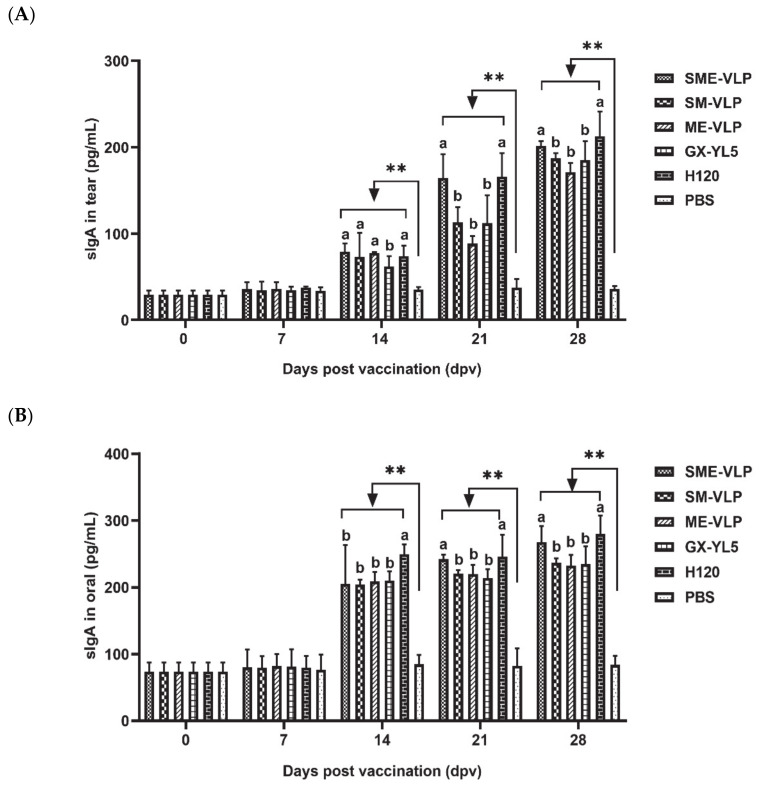
sIgA concentrations in tear and oral swabs at 0, 7, 14, 21 and 28 dpv. (**A**) sIgA concentrations in tear swabs. (**B**) sIgA concentrations in oral swabs. Letters (a,b) indicate significant differences among vaccinated groups (*p* < 0.05). ** indicates *p* < 0.01 between vaccinated and nonvaccinated groups. (*n* = 10 chickens/group.)

The sIgA levels were detected in tear and oral collections at 0, 7, 14, 21 and 28 dpv (Figure 10). The sIgA levels of the tear and oral collections from the vaccinated chickens were significantly higher (*p* < 0.01) compared to the negative control group from 14 to 28 dpv. At 14 dpv, the H120 group had higher sIgA levels in the oral swabs than the other immunized groups (*p* < 0.05). At 21 and 28 dpv, the SME-VLP and H120 groups had higher levels of sIgA in the tear and oral swabs than the SM-VLP, ME-VLP and GX-YL5 groups (*p* < 0.05), but no significant differences between the SME-VLP and H120 groups were apparent. Furthermore, at 21 and 28 dpv, no significant differences were observed among the SM-VLP, ME-VLP and GX-YL5 groups.

### 3.6. Protection against the IBV Challenge

**Figure 11:**
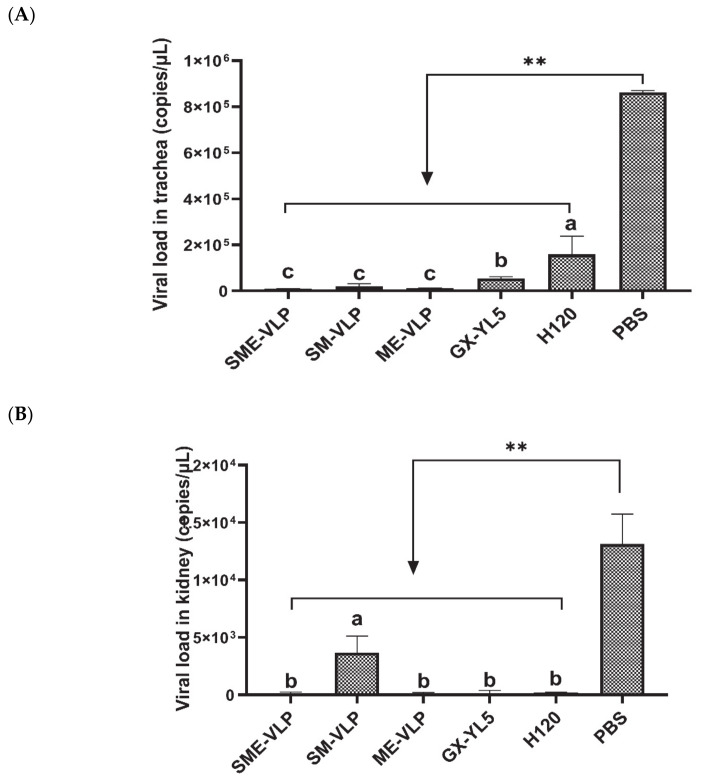
Viral loads in tracheas and kidneys. IBV RNA levels in tracheas and kidneys were evaluated at 5 days post-challenge (dpc). (**A**) Viral loads in tracheas. (**B**) Viral loads in kidneys. Letters (a–c) indicate significant differences among vaccinated groups (*p* < 0.05). ** indicates *p* < 0.01 between vaccinated and nonvaccinated groups. (*n* = 10 chickens/group.)

Under the IBV strain GX-YL5 challenge, no apparent symptoms were observed in the vaccinated birds. The birds in the negative control group showed depression, gasp, tracheal rales and watery eyes in the early days of the challenge. At 5 dpc, all the vaccinated birds had significantly lower viral RNA levels in the tracheas and kidneys compared to the negative control (Figure 11). The viral RNA levels in the kidneys of the immunized groups were lower than those in the tracheas. Among all the vaccinated groups, the viral loads in the tracheas of the H120 group were significantly higher than those of the four other immunized groups (*p* < 0.05). According to the viral RNA levels in the tracheas, the immunized groups ordered from lowest to highest were the SME-VLP group, ME-VLP group, SM-VLP group, GX-YL5 group and H120 group, but no significant differences were observed among the three VLP groups. The viral RNA levels in the kidneys of the SME-VLP, ME-VLP, GX-YL5 and H120 groups were significantly lower than those in the SM-VLP group (*p* < 0.05), but no significant differences were noted among the SME-VLP, ME-VLP, GX-YL5 and H120 groups. 

**Figure 12:**
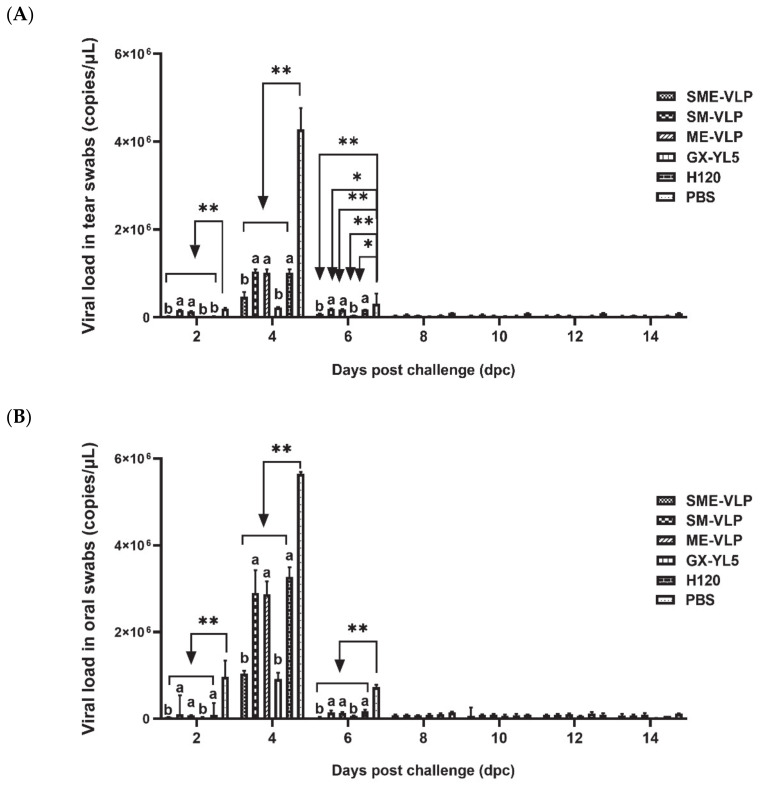
Viral loads in tear and oral swabs. IBV RNA levels in tear and oral swabs were evaluated at 2, 4, 6, 8, 10, 12 and 14 dpc. (**A**) Viral loads in tear swabs. (**B**) Viral loads in oral swabs. Letters (a,b) indicate significant differences among vaccinated groups (*p* < 0.05). ** and * indicate *p* < 0.01 and *p* < 0.05 between vaccinated and nonvaccinated groups, respectively. (*n* = 10 chickens/group.)

The virus shedding in the tear and oral swabs was monitored after the IBV challenge. The results show that the vaccinated birds had significantly lower viral loads compared to the negative controls from 2 to 6 dpc (*p* < 0.05 or *p* < 0.01) (Figure 12). The viral RNA was detected in the tear and oral swabs at 2 dpc and reached a peak at 4 dpc, and then decreased at 6 dpc, thereafter remaining at very low levels until 14 dpc. At 2 dpc, the viral RNA levels in the tear swabs of the SME-VLP, GX-YL5 and H120 groups were significantly lower than those in the SM-VLP and ME-VLP groups (*p* < 0.05). At 4 and 6 dpc, the viral RNA levels in the tear swabs of the SME-VLP and GX-YL5 groups were significantly lower than those of the three other immunized groups (*p* < 0.05), but no significant differences were noted between the SME-VLP and GX-YL5 groups. At 2, 4 and 6 dpc, the viral RNA levels in the oral swabs of the SME-VLP and GX-YL5 groups were significantly lower than those in the SM-VLP, ME-VLP and H120 groups (*p* < 0.05), and no significant differences were observed between the SME-VLP and GX-YL5 groups. 

**Figure 13:**
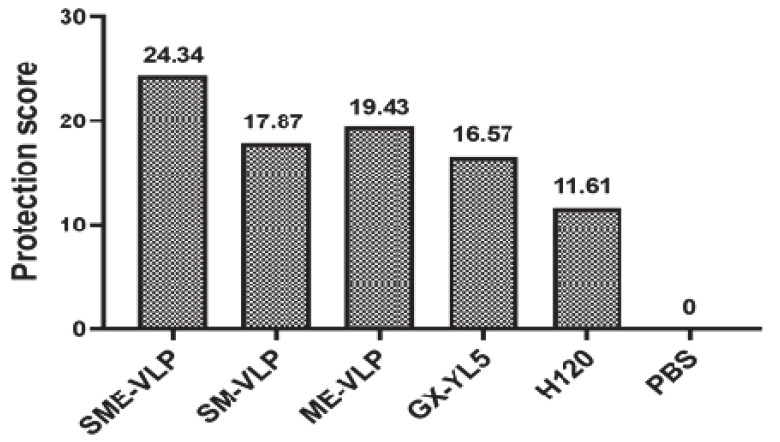
Ciliostasis protection scores for tracheas in challenged chickens at 5 dpc (*n* = 10 chickens/group).

At 5 dpc, the ciliostasis of each group was measured, and the protection scores were determined (Figure 13). The higher the protection score, the better the protective efficacy of the vaccination program. The groups sorted by protection scores from highest to lowest are as follows: the SME-VLP group, ME-VLP group, SM-VLP group, GX-YL5 group, H120 group and negative control group. All the protection scores of the SME-VLP, ME-VLP and SM-VLP groups were higher than those of the GX-YL5 and H120 groups. Among the three VLP-vaccinated groups, the SME-VLP group had the highest score (the protection score was 24.34). The nonvaccinated group failed to be protected (the protection score was 0). 

## 4. Discussion

IB is a highly contagious disease and brings serious economic losses to the poultry industry. Today, live-attenuated and inactivated vaccines are frequently used to control and prevent this disease in commercial chickens. However, the current commercial vaccines have defects in safety or immunogenicity [46]. Live-attenuated vaccines are not effective in stimulating humoral responses and can cause the emergence of novel variants due to mutation and recombination [21,47]. Inactivated vaccines can stimulate humoral immunity but very low levels of cellular and mucosal immunity responses [48,49]. At present, there are dominant serotypes of IBV strains in the field, although multiple serotypes have coexisted [25,27]. The dominant serotypes of IBV field strains are not consistent with those targeted by the commercial vaccines. Therefore, the commonly used vaccines cannot provide adequate protection against the endemic strains. Hence, it is very urgent to develop an effective new vaccine using the prevalent strain of dominant serotypes to offset the immune deviation from traditional vaccines. This study was designed to construct and compare the immunogenicity of three IBV VLPs carrying different combinations of structural proteins based on the dominant serotype representative strain GX-YL5 in southern China, providing a vaccine candidate for IB. To our knowledge, it is the first report on the construction and immunogenicity comparison of three VLPs carrying different combinations of structural proteins of the IBV dominant epidemic strain in southern China.

In this study, SME-VLPs, SM-VLPs and ME-VLPs, carrying the S, M and E proteins, the S and M proteins, and the M and E proteins, respectively, of the dominant serotype representative strain GX-YL5 in southern China were constructed. All the results of Western blotting, TEM and IEM proved that the SME-VLPs, SM-VLPs and ME-VLPs were successfully assembled. As far as we know, IBV VLPs were observed by IEM for the first time in our study. In addition, we firstly systematically investigated the humoral, cellular and mucosal immune responses to different combinations of IBV VLP vaccines. Among the three VLPs, it was found that the SME-VLPs could induce neutralizing antibodies equivalent to those induced by the inactivated GX-YL5 vaccine and higher than those with the attenuated H120 vaccine; the percentages of CD4^+^ and CD8^+^ T lymphocytes, and IL-4 and IFN-γ concentrations were higher than those observed with the GX-YL5 inactivated vaccine; the sIgA levels of the tear and oral fluids were equivalent to those seen with the attenuated H120 vaccine and higher than those with the inactivated GX-YL5 vaccine. In the challenge study, the SME-VLPs induced significantly lower viral RNA levels in the trachea and higher protection scores than those of the GX-YL5 and H120 groups, viral RNA levels in the kidneys and tear and oral swabs comparable to those in the GX-YL5 group, and significantly lower virus shedding in the tear and oral swabs compared to the H120 and negative control groups. Hence, our results suggest that among the three VLPs, the SME-VLPs could efficiently stimulate the vaccinated birds to generate the strongest humoral, cellular and mucosal immune responses, and showed good protective efficacy against virulent IBV challenges, indicating that it would be useful as an appealing vaccine candidate for the development of IB vaccines in the future.

The VLPs carrying the M and S proteins of the H120 strain induced comparable humoral immune responses and a higher cellular immune response than the inactivated H120 vaccine [38]. Another VLP carrying the S, M and E proteins of the M41 strain could also elicit IBV-specific antibodies and neutralizing antibodies comparable to those induced by the inactivated M41 viruses [39]. In our study, the IBV-specific antibody levels in the three VLP groups were lower than those of the GX-YL5 and H120 groups. However, the neutralizing antibody in the SME-VLP group was comparable to that of the inactivated GX-YL5 group, and higher than those of the SM-VLP, ME-VLP and H120 groups, which was not consistent with the IBV-specific antibody results. A possible reason is that the coated antigen of the commercial ELISA IBV antibody test kit was for the Mass-type virus strain, and the GX-YL5 strain used in our study was a serotype heterologous to the Mass-type virus strain [25]. In addition, the GX-YL5 and H120 vaccines were based on the whole virus and comprised almost all the epitopes of IBV. Therefore, they proved better in the activation of IBV-specific antibody responses. Another reason could be that the immune dose of 2 μg/bird and immunization times might not have been enough [42]. A previous description reported that chimeric IB-ND VLPs (100 μg of proteins) produced antibody levels comparable to those that adjuvanted inactivated vaccines did [41]. Chickens in the VLP groups had slightly higher neutralizing antibody levels than the M41 group [41], which is in agreement with our observation. The neutralizing antibody level is a key criterion of vaccine efficacy evaluation. The high neutralizing antibody level induced by SME-VLPs proved that they have the potential to be a vaccine candidate. 

Additionally, our study showed that SME-VLPs could induce significantly higher percentages of CD4^+^ and CD8^+^ T lymphocytes than the inactivated GX-YL5 viruses, comparable to those of the H120 group. A previous study showed that rHBM-S1-N and rHBM-N generated higher CD4^+^ and CD8^+^ T lymphocyte percentages compared to those induced by a H120 vaccine [41]. In another study, the recombinant BacMam virus could generate a greater antigen-specific T cell response [50]. IFN-γ and IL-4 are markers for T helper type 1 (Th1) and Th2 responses, respectively. SARS-CoV VLPs formed from the S, E and M proteins induced a Th1-based cellular immune response in mice immunized intraperitoneally but not in mice immunized intranasally [33]. MERS-CoV VLPs containing the S, E and M proteins could induce an increase in IFN-γ but not in IL-4 [34]. However, IBV VLPs carrying the M and S proteins of the H120 strain induced significantly higher IL-4 and IFN-γ levels than the inactivated H120 vaccine [38]. A recent study also revealed that chimeric IB-ND VLPs from the IBV M41 strain and NDV La Sota strain induced significantly higher IL-4 and IFN-γ levels than the M41 and La Sota inactivated vaccines [41]. Our study showed that the IL-4 and IFN-γ levels from the SME-VLPs were significantly higher than those from inactivated GX-YL5. Therefore, our SME-VLPs could evoke both Th1 and Th2 cellular immune responses, which was similar to the findings of previous IBV VLP studies [38,41].

IBV is a respiratory pathogen and firstly enters the body through the respiratory tract. For vaccines for respiratory disease, it is very critical to stimulate the host to produce mucosal immune responses in addition to the systemic immune responses. Past findings showed that sIgA was detected in mucosal secretions and tissues in mice immunized intranasally but not in mice immunized intraperitoneally with SARS-CoV VLPs formed from the S, E and M proteins [33]. To date, no previous studies on the mucosal immune response to IBV VLP vaccines have been reported. In this study, we were the first to analyze the mucosal immune response to VLP vaccines for IBV. Our results reveal that intramuscularly administered SME-VLPs could induce sIgA at a level equivalent to that induced by the H120 live-attenuated vaccine and higher than those achieved with SM-VLPs, ME-VLPs and the inactivated GX-YL5 vaccine, showing that the SME-VLP vaccine had a good ability to stimulate mucosal immunity. The strong mucosal immunity induced by SME-VLPs proved, again, that they would be a promising vaccine candidate.

In the challenge study, the vaccinated birds had significantly lower viral RNA levels in the tracheas and kidneys, and significantly lower virus shedding in the tear and oral swabs compared to the nonvaccinated birds, and the VLP-vaccinated birds had higher protection scores, indicating that the three VLPs could provide protection against the IBV challenge. At 5 dpc, the SME-VLP group had the lowest viral RNA levels in the tracheas, which were significantly lower than those with the GX-YL5 and H120 vaccines; the viral RNA levels in the kidneys of the SME-VLP and ME-VLP groups were significantly lower than those of the SM-VLP group. From 2 to 6 dpc, the SME-VLP and GX-YL5 groups demonstrated significantly lower virus shedding in the tear and oral swabs compared to the SM-VLP and ME-VLP groups, and no significant differences were observed between the SME-VLP and GX-YL5 groups. The SME-VLP group had the highest protection scores compared to the SM-VLP and ME-VLP groups. Therefore, collectively, the SME-VLPs provided better protection against the IBV challenge than the ME-VLPs and SM-VLPs, especially in reducing the shedding of the virus in the tear and oral swabs in early infection, which was consistent with the systemic and mucosal immune responses induced by the SME-VLPs. In addition, our results show that the SME-VLPs provided better protection in the trachea compared to the inactivated GX-YL5 vaccine, which may be due to the higher cellular immune response and mucosal immune responses compared with those elicited by the GX-YL5 inactivated vaccine, which agrees with our previous study [42]. In the future, the protection against a challenge with a heterologous IBV strain will be observed.

Many factors affect the immune response, such as the route of administration of an immunogen, frequency, dose, time interval and animal health. A previous report showed that higher sIgA was detected in mucosal secretions and tissues in mice immunized intranasally than those immunized intraperitoneally with SARS-CoV VLPs [33]. In our study, although the birds were intramuscularly immunized with the SME-VLPs, excellent systemic and mucosal immune responses were induced, especially cellular and mucosal immune responses. Therefore, the SME-VLPs had good immunogenicity. Of course, long-term immunity is very important for a vaccine. In our study, due to the limitations of objective conditions, the observation time for immunity was not long enough. Additionally, the egg production could not be tested in the vaccinated birds. Increasing the observation time for immunity and testing egg production will be conducted in a near-future clinical trial. In addition, the SME-VLPs will be assembled for different serotype strains and then mixed together to prepare multivalent inactivated vaccines in the future. The SME-VLPs can also be formed from the S proteins, M proteins and E proteins from different strains. Additionally, the baculovirus expression system can easily be scaled up from the laboratory to mass production [42]. Intranasal immunization will also be attempted as a mass immunization strategy for SME-VLPs in the future. 

## 5. Conclusions

In summary, SME-VLPs, SM-VLPs and ME-VLPs, which carried the S, M and E proteins; the S and M proteins; and the M and E proteins, respectively, were firstly constructed and compared for immunogenicity in the present study. Among the three VLPs, the SME-VLPs could stimulate the strongest humoral, cellular and mucosal immune responses, and provide efficient protection against a virulent IBV challenge. To our knowledge, our study showed, for the first time, that IBV VLPs were identified by IEM. In addition, the humoral, cellular and local mucosal immune responses to and immunogenicity of different combinations of IBV VLP vaccines were systematically investigated for the first time. This work suggests that the SME-VLPs could serve as an appealing vaccine candidate for the IB vaccine.

## Data Availability

The data presented in this study are available on request from the corresponding author.
